# Supplementary material for: Apple Glycosyltransferase MdUGT73AR4 Glycosylates ABA to Regulate Stomatal Movement Involved in Drought Stress
Source: Int J Mol Sci. 2024 May 23;25(11):5672. doi: 10.3390/ijms25115672 (PMC11171509; doi:10.3390/ijms25115672)

Figure S1 Schematic representation of the construction of the recombinant vector MdUGT73AR4-pGEX-2T.

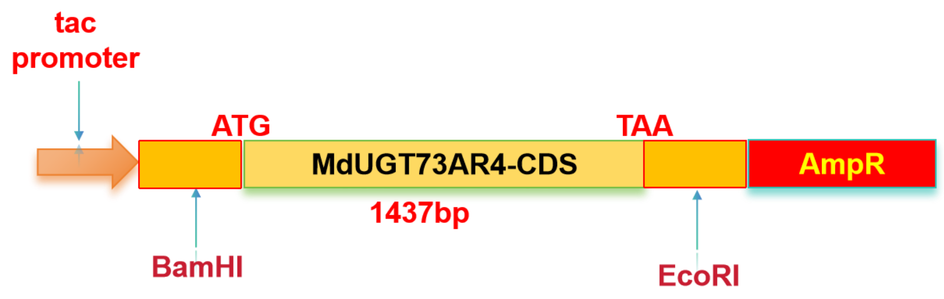

Figure S2 Schematic diagram of the construction of recombinant vector MdUGT73AR4-PBI121.

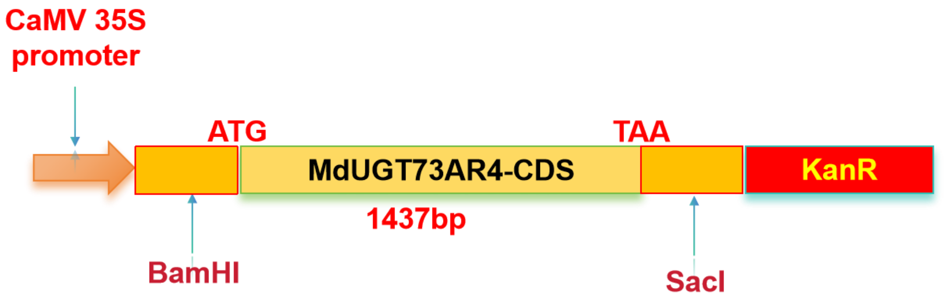

Figure S3 EMSA experiments demonstrate that *MdAREB1B* binds most tightly to the *MdUGT73AR4* proximal AREB regulatory element (369bp from the *MdUGT73AR4* start codon ATG).

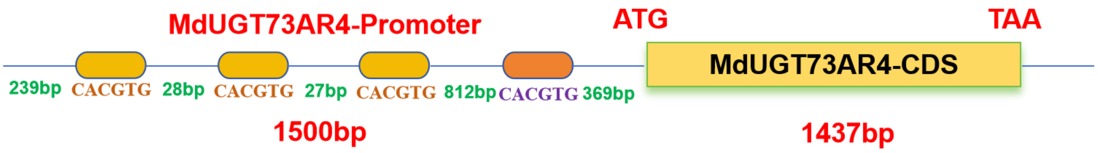

Supplement: Supplementary file 1 [file ijms-25-05672-s001.zip › MdUGT73AR4 Supplement Figure.pdf]
