# Supplementary material for: Apple Glycosyltransferase MdUGT73AR4 Glycosylates ABA to Regulate Stomatal Movement Involved in Drought Stress
Source: Int J Mol Sci. 2024 May 23;25(11):5672. doi: 10.3390/ijms25115672 (PMC11171509; doi:10.3390/ijms25115672)
Supplement: Supplementary file 1 [file ijms-25-05672-s001.zip › MdUGT73AR4 Supplement Table.pdf]

Table S1 Gene Primers

Gene name	Primer Sequences	Function
qMdUGT73AR3	F: ACAGCGTTTGGGAGGAAG<br>R: TGCCTAGAAGCCTCAAGC	For real-time PCR
qMdUGT73AR4	F: GCTAGCTTAGTTACCACC<br>R: CAGCTTCGCGTGAGGCTC	For real-time PCR
qMdUGT3AR5	F: GCTTCTGGGCAGGAGTTC<br>R: AAACTGCTCAGCCGACAC	For real-time PCR
qMdUGT3AR6	F: ACGCGAAGCTGTCATCT<br>R: CTATATGCCATGCCTTCC	For real-time PCR
qMdUGTAB13	F: CTACCTTCGCGAGCAT<br>R: GAGCCTTAGTGATCTC	For real-time PCR
qMdUGT73AC7	F: GCATCGTAATCGACGTC<br>R: TGGCCTGTTTCTTGCG	For real-time PCR
qMdSPR1	F: ACCTCCGAGTTGATGGC<br>R: TCAGTTCCCACTCCCAC	For real-time PCR
qMdSPR2	F: GAAACCACCTGTCTTAACTG<br>R: CGTGTCTACGAGGAACAACTA	For real-time PCR
qMdTUB1	F: AGTGGCGTAACTTGCTGC<br>R: GTCAACTTCCTTGGTGCT	For real-time PCR
qMdMAP65-1	F: GATAGCGAAAGCGAATG<br>R: ATCTTCCTCCCAGCCAC	For real-time PCR
qMdMAP65-3	F: CTTTCAATGCAAGCTCCTA<br>R: AACATCCAAGCCTCGTC	For real-time PCR
qMdMAP65-5	F: AATGACCAGGAAGCACAG<br>R: AACAGGTTTGAGGGCAGA	For real-time PCR
qMdMAP65-6	F: TGCTCCAGTCAGTTCGTT<br>R: GATCCACTTCACCCACAG	For real-time PCR
qMdAREB1B	F: TATGGACTCGTGTCGAGGCGGC	For real-time PCR

	R: AAATATGACTTGTTTAATTAAAC	
qMdUBQ	F: CTCCGTGGTGGTTTTTAAGT<br>R: GGAGGCAGAAACAGTACCAT	For real-time PCR
cMdUGT73AR4	F: CGAGTATCGACCGTGAATATGG<br>R: TCATGCTCCAAGGGACCCCAAT	For amplifying full-length cDNA
cMdAREB1A	F: GTTTCACAGAAAAATGGGGACC<br>R: CGATTGAGGATTCGTTCATTAAC	For amplifying full-length cDNA
cMdAREB1B	F: TATGGACTCGTGTCGAGGCGGC<br>R: AAATATGACTTGTTTAATTAAAC	For amplifying full-length cDNA
cMdAREB2B	F: TTTCTGCATATTCGACGCCTG<br>R: TCATCGTTCTTAACAAGCTCAC	For amplifying full-length cDNA
cMdAREB2A	F: TTTCTGCATTCGATCAACTTGG<br>R: GAACTGACACAATCGATTCAC	For amplifying full-length cDNA
Pro-UGT73AR4	F:GCTGGTAACCTCGACCTCACCCAGGCC TTCTTCACCACGTGCGTTCTGGC<br>R: CGACCATTGGAGCTGGAGTGGGTCCG GAAGAAGTGGTGCACGCAAGACCG	Synthesized probe surrounding proximal DRE element
MPro-UGT73AR4	F: GCTGGTAACCTCGACCTCACCCAGGC CTTCTTCACTCATGACGTTCTGGC<br>R: CGACCATTGGAGCTGGAGTGGGTCCG GAAGAAGTGAGSTACTCGTTCTGGC	For introducing mutation to promoter elements
Pe-DREB1B	F: TATGGACTCGTGTCGAGGCGGC<br>R: AAATATGACTTGTTTAATTAAAC	Protein expression in *Ecoli*
ChIP-UGT73AR4	F: GCTAGCTTAGTTACCACC<br>R: CAGCTTCGCGTGAGGCTC	For amplifying fragments in ChIP

Table S2 Full-length sequence of MdUGT73AR4 nucleotides

ATGGAAACTAAATCCCATAAGCAGCTTCACATTTTCTTCTTCCCATATATGGTTCAAGGC<br>CACTTCGTACCCCTTATAAACATTGCCAGACTATTTGCTTCTCGTGGTGTTAAATCCACC<br>CTAATAGCCACCCCTCTCAATGAACCTCTCTTTTCCAAGGCAATCCAAAGCAGCAAGA<br>AATTAGGCTTTGATGTTGACATTCTTGTCATCAAGTTCCCAGCTGAGGAAGTAGGTTTG<br>CCTCAAGGATGTGAAAATGCTAGCTTAGTTACCACCACGGAGATGAACGAAAAGTTCA<br>TCAAAGCCACCTTCCTTCTTCAACCACAAATTGAGCAGATTTTAGACAAACACCGCCC<br>TCATTGCCTTGTTGCAGACACGTTCTTTCCTTGGGCAACGGAAGTTGCTGCCAAGTTTG<br>ATATTCCCAGGATCATATTTCATGGCATGGGTTTTTCGCTTTGTGTGCTTCTCGTAGTGT<br>GGCGTTTGTGGCGTTGTATGAGCCTCACGCGAAGCTGTCATCTGATTCAGAAGTTTTA<br>CTATTCCTAGTTTTCCAGTTGAGATCAAGCTGACAAGAAGCCAAATCCCGAATTTTCCC<br>AAGCAAAGTGCTGAATTCACCAAGTTGTTTAAAGAGGCGATGGAGAGCGAGGAAAAG<br>AGCTATGGGTTCATTGTTAACAGTTTCTATGAACTTGAACCGGCTTTTGCAGACCATTA<br>CAGGACAGTGTTGGGGAGGAAGGCATGGCATATAGGCCCGGTTTCGTCAGTCAATAAG<br>GCAGCAGATGACGAAGCCTTCCTCGATCGGCACGAGTGCTTGAATTGGCTTAGTTCTA<br>AGAAACCCAATTCAGTTGTTTACATATGTTTCGGAAGTATGACCAAATTCATTGACTCTC<br>AGCTCCTAGAAATTGCAGCGGGGCTTGAGGCTTCTGGGCAGGAGTTCATTTGGGTTGT<br>GAAGAGAGAAAAGAACGATAAAGAAGAGTGGCTCCCCGAAGGGTTTGAGAAGAGAA<br>TGGAAGGTAAAGGACTAATTATAAGAGGTTGGGCTCCGCAAGTGCCGATTCTTGAGCA<br>CCAAGCAATCGGAGCCTTCGTGACTCACTGCGGGTGGAACTCTATCCTTGAAGGAGTA<br>TCTGCTGGGGTACCAATGATCACATGGCCCGTGTCGGCTGAGCAGTTTTACAATGAGA<br>AGTTGGTGACCGTGGTACTGAAAACTGGGGTTGCTGTTGGTGCTAAACAATGGGGTAC<br>ACATCTGGATGTGATGACGGAAGCCAGTGTGAAGAGGGAAGCCATAGAAAAGGCTGT<br>AAATCAAGTGATGGTGAGTGAAGAAGCAGAGGGAATGAGAGGCAGAGCCAGGATGCT<br>TAGAGAGATGGCAATGAGGGCTGTTGAAGAAGGTGGTTCGTCTTTCTCAGATTTAACT<br>TCTCTAATTCAGGAATTGGGGTCCCTTGGAGCATGA

Table S3 Amino acid sequence of MdUGT73AR4

METKSHKQLHIFFFPYMVQGHFVPLINIARLFASRGVKSTLIATPLNEPLFSKAIQSSKKLGF
DVDILVIKFPAEEVGLPQGCENASLVTTTEMNEKFIKATFLLQPQIEQILDKHRPHCLVADTF
FPWATEVAAKFDIPRIIFHGMGFFALCASRSVAFVALYEPHAKLSSDSEVFTIPSFPVEIKLTR
SQIPNFPKQSAEFTKLFKEAMESEEKSYGFIVNSFYELEPAFADHYRTVLGRKAWHIGPVSS
VNKAADDEAFLDRHECLNWLSSKKPNSVVYICFGSMTKFIDSQLLEIAAGLEASGQEFIW
VVKREKNDKEEWLPEGFEKRMEGKGLIIRGWAPQVPILEHQAIGAFVTHCGWNSILEGVS
AGVPMITWPVSAEQFYNEKLVTVVLKTGVAVGAKQWGTHLDVMTEASVKREAIEKAVN
QVMVSEEAEGMRGRARMLREMAMRAVEEGGSSFSDLTSLIQELGSLGA

Table S4 Sequence of the MdUGT73AR4 promoter

ATAAGACAGATAAGGCAAGTGAAGATGATACCACACTTCGATAGTTAGAAGTTTTATGA
TTACTCAGCAACTTGGATCTTGCAAGTCCCCAACCGAGGAGCTTCCCTCACTCGAAAA
CTTAAGGGAACACTGTTTGTACCACACTTGACCAATCTCGAAACTACTAAGCACCGGT
CAAAGTTATACCGTTAAGGATCCAAAAGAGTTTCCCTCCGACCAGGAGGCCAATCACA
ATGCGACACGTGTTGACATCAGAAGCCAATCACAGCGCGACACGTGTCAACATCAGA
AGCCAATCATAACATGACACGTGTCAATGTCAAAACAAAGCTAAAAACTCTTCTATAA
AATGAGATCATTCTCCCACAATATGGCCTAATGTCATTTGTACTAAATCATTCACTAGTA
CTCACAAAATGAGAGCTTAAACATATGTACTTGTGTAAACCCTTCACAATTAATGAGAA
CTTATCTGCTCCGTGGACGTAGCCAATATGGGTGAACCACGTACATCTTGGTTTGCTTCA
TTGTCTCTATCCATTTACATACTTATCCACACTAGTGACCGGAGCAATCTAGCGAAGGTC
ACAAACTTGACACTTTCTGTTGTATCAAAGTCCTCACTGATTTTGTGCATCAACATTTG
GCGTCGTCTATGGGAATCAGCACGAAAAACTATGTCGGTTCTCTTTCATTTTTTTCATTT

CACCACCGTGAAACCTCATCACAACCTCCACCGTGAATCTGTAGAAACCCAAGAACCA
AACAACCCAACACCCACACTCAGAGACACACCCATTCATTCTATAGAGAGAGAGACTC
AGATAAATCCCTATTTTCTTTTCTTTTTTTTCAATGAGATCTTCATATTCTTCTTCAACAAT
GTTTGCTTTGTAAAATCCAAAAGTTTTTATTTGTGTTTTCTCATCCATTTTGTGAAATTTT
CTTTCTTTTTCTCTTTCTGGAAACATAAAATCAAAACCCTTGTCGGGCGAGTCAACGAC
CGAGTCGGGGGGACTCTGTCGAGTCAGAGATCGAGGGTGGTGATAGGAAAGAAGTGG
GTGGGCAGAGAAAATATCTCTCCCCGCAAGCTGGTAACCTCGACCTCACCCAGGCCTT
CTTCACCACGTGCGTTCTGGCAAAAAGTTTATCAGAAAGGTCAGAATTCTCACCATCG
ACCCCACCGGAGCCACCGATGGCATCTTCACCGCCAATGCTCTCTCCCACCTCGAGTC
CTCTCTCCGTCAAAAATTTGGTGACCCCAACGCCGCCATCTCTAACTTATTCAACGTCG
TTTCCAAGTCAAGTGATGGCGGCATTCTCGTCGCCCTCCTCTTCACTCGAGGTTTAAAG
GAAGGGTCGACTTGACCCATGTTCATCGCCAAAAAAGCCCTTCAAACCATATCCGGAT
GGGGTTCCCCAGTGGGTTTTTAGGCCATTGAAGGCTGAGAAAGAGAGGGAGAAACTC
TTTCCGAACACGTTCGAGTATCGACCGTGAAT
